# Effectiveness of purple leaves (Graptophyllum pictum L. Griff) and hydroxyapatite as socket preservation biomaterials

**DOI:** 10.1016/j.jobcr.2024.12.002

**Published:** 2024-12-09

**Authors:** Ratri Maya Sitalaksmi, Rizkipriyanto Azharpratomo, Shafira Ninditya Irsan, Primanda Nur Rahmania, Sisca Meida Wati, Syafira Salsabila Adam, Kavanila Bilbalqish, Khairul Anuar bin Shariff, Harry Laksono, Agus Dahlan, Devi Rianti

**Affiliations:** a Faculty of Dental Medicine, Universitas Airlangga, Indonesia; b Magister Graduate Student of Dental Health Science, Faculty of Dental Medicine, Universitas Airlangga, Indonesia; c Resident of Prosthodontics, Faculty of Dental Medicine, Universitas Airlangga, Indonesia; d Department of Prosthodontics, Faculty of Dental Medicine, Universitas Airlangga, Indonesia; e Department of Oral and Maxillofacial Pathology, Faculty of Dental Medicine, Universitas Airlangga, Indonesia; f School of Material and Mineral Resources Engineering, Engineering Campus, Universiti Sains Malaysia, Malaysia; g Department of dental materials, Faculty of Dentistry Medicine, Universitas Airlangga, Indonesia

**Keywords:** Socket preservation, Hydroxyapatite, Purple leaves, Bone regeneration

## Abstract

**Background:**

Tooth extraction without socket preservation will lead to reduction in the dimensions and volume of the alveolar bone. Bone defects resulting from tooth extraction not only hinder prosthetic reconstruction but also present aesthetic issues and complicating dental implant treatment. Purple leaves contains flavonoids, steroids, tannins, saponins,and non-toxic alkaloids. These compounds play roles as antimicrobials, immunomodulators, antioxidants, anti-inflammatories, analgesics. Hydroxyapatite has good biocompatibility, and can induce osteoblast differentiation.

**Aim:**

To investigate the effects of the combining nanoemulsion extract of purple leaves and hydroxyapatite on the expression of RUNX2, OSX, OPN, ALP, and calcium deposition.

**Method:**

This research used MTT Assay, ICC, and Alizarin Red staining. The study groups were as follows: Group 1: Ad-MSCs; Group 2: Ad-MSCs + αmem; Group 3: Ad-MSCs + osteogenic medium + 1 % nano-extract of Purple leaves & Hydroxyapatite; Group 4: Ad-MSCs + osteogenic medium + 2 % nano-extract of Purple leaves & Hydroxyapatite. Observations were conducted on days 7, 14, and 21.

**Results:**

The combination of nanosuspension extract of Purple leaves and hydroxyapatite significantly increased the expression of RUNX2, OSX, OPN,ALP and calcium deposition compared to other groups. The combination of nanoemulsion extract of Purple leaves and hydroxyapatite were significant (P < 0.05) compared to the control group on each day 7, day 14 and day 21.

**Conclusion:**

The combination of nanosuspension extract of Purple leaves and hydroxyapatite was able to enhance the expression of RUNX2, OSX, OPN, ALP, and calcium deposition on days 7,14,21.

## 1 Introduction

Tooth extraction initiates a series of events that result in significant changes in the height and width of the alveolar ridge. Post-extraction socket preservation is a procedure that can be performed to reduce alveolar bone resorption. Extraction without socket preservation poses the ridge will undergo atrophy, the peak volume of the alveolar ridge decreases, particularly during the first 6 months and mostly on the buccal wall.^1^ Socket preservation falls within the realm of bone tissue engineering (BTE). The gold standard is autografting, that involves taking donor tissue from the individual and applying it to the existing bone defect. Through the combination of stem cells, scaffolds, and growth factors (GFs), BTE achieves biomimetic conditions to enhance tissue and cell regeneration and growth.^2^

Socket preservation can help maintain residual ridge, thus providing a high success rate for implant and fixed prosthesis treatments. Hydroxyapatite (HA) is a commonly used material in socket preservation as a bone graft. HA is the most stable calcium phosphate compound in terms of temperature, pH, and composition in the bloodstream, and its a derivative of calcium phosphate with a chemical formula and properties similar to the inorganic minerals found in bone and teeth.^3^ HA has been shown to have good Biocompatibility and osteoconductive properties, meaning it can be well tolerated by human oral cavity tissues and can stimulate osteoblast differentiation. HA also has the ability to induce mesenchymal cells to differentiate into osteoblasts, making it a suitable scaffold material for bone tissue engineering.^4^

Indonesia, as a tropical country, boasts a rich biodiversity. Through the Indonesian Food and Drug Monitoring Agency (BPOM), Indonesia has highlighted several indigenous medicinal plants.^5^ Herbal medicine comprises numerous molecules that synergistically act on specific cellular targets. Purple leaf (Graptophyllum pictum Griff) belonging to the Acanthaceae family, has been registered as a medicinal plant in the pharmacopoeia's second edition in 2017 and used in traditional medicine to treat various diseases.^6^ Purple leaf contains non-toxic alkaloids, flavonoids, steroids, saponins, and tannins, these constituents play roles as antimicrobials, immunomodulators, antioxidants, anti-inflammatory, analgesic, wound healing agents, and others.^7^ Flavonoids also influence immune cells and mechanisms involved in inflammation processes. In a previous study purple leaf was found to stimulate ALP activity by 128 % in MC3T3-E1 osteoblast cells and also has the potential to reduce the number of osteoclast cells in Wistar rats induced by P. gingivalis with optimal concentrations of 5 % and 10 %.^8^

Alkaline Phosphatase (ALP) is an ectoenzyme that hydrolyzes ester monophosphates and it is widely used in research as an early marker of osteoblast differentiation. The expression of ALP decreases followed by an increase in late markers such as osteocalcin (OCN). Meanwhile, HA simultaneously downregulates the ALP gene and upregulates osteopontin (OPN), OCN, and COL1.^9^ This process is followed by calcium and phosphate deposition.^10^ Cells such as osteoblasts and ASCs that have osteogenic properties will usually produce calcified nodules that adhere to the culture plate.^11^ Osteoblast differentiation is a complex process involving the transcription factor osterix (Osx) for osteoblast differentiation and bone formation.^4^ Osteopontin (OPN) is a sialoprotein expressed by several cells including osteoblasts, osteocytes, and odontoblasts. The expression and upregulation of osteopontin are influenced by transcription factors including Runt-related transcription factor 2 (Runx2) and Osterix.^12^ Runx2 is a key transcription factor expressed by osteoblast lineage cells and chondrocytes. Precursor osteoblasts expressing Runx2 are called preosteoblasts. Runx2 is a major marker studied in osteoblast differentiation, particularly in the early differentiation phase. It has been proven that the use of biomaterials such as HA can enhance osteoblast differentiation by upregulating Runx2.^9^

Combining purple leaf and HA, each with their respective constituents, is expected to yield a material suitable for bone tissue engineering. Nanobiotechnology has advanced, and nano-sized biomaterials have been widely applied for tissue engineering.^13^ Various nano-structured matrices have been shown to stimulate cell differentiation with a focus on maintaining structural, compositional, and biological features of bone tissue. The main constituent used so far in this regard is nano-hydroxyapatite.^14^ This research proves that the combination of nano suspension from purple leaves and hydroxiapatite in vitro can increase the bone remodeling process so that it is concluded that it can be a candidate for use as therapy in socket preservation as a preparation for supporting tissue for dental implants.

## 2 Methods

### 2.1 Etik clearance

This study has been approved by ethical health committee of the Faculty of Dental Medicine, Universitas Airlangga with the number of certificate No: 0673/HRECC.FODM/VII/2024, 1390/HRECC.FODM/XII/2023, and 1410/HRECC.FODM/XII/2023. This study design uses the laboratory experiment with a post-test-only control group design.

### 2.2 Purple leaves extraction process

Purple leaves extraction process in the Biology Department Universitas Katolik Widya Mandala, Surabaya, Indonesia. Purple leaves 600 g were subjected to an extraction process involving 2 L of 96 % ethanol and were keep at room temperature and placed in a closed container for 3 days and filtered with filter paper to obtain macerate. The pulp was evaporated to obtain a thick extract of purple leaves.

### 2.3 Nanosuspension preparation

Nanosuspension was made by adding 10 ml of hot distilled water to the mortar, then adding 1 g of CMC Na and waiting for 15 min. Next, stir until it became a gel mass. Then 1 g of nipagin solution dissolved in 10 ml of distilled water was added to the gel mass. The extract solution (1 g) that has been dissolved in 20 ml of 96 % ethanol was stirred until homogeneous in mortar. Hydroxyapatite (nanoXIM®HAp200, FLUIDINOVA, Portugal). Next, 100 g of distilled water was added and Turax for 10 min. The nanosuspension was stirred at a speed of 1400 rpm for 90 min at 50 °C.

### 2.4 Toxicity test with MTT assay

The MTT assay is a sensitive, quantitative and reliable colorimetric assay aimed at assessing the toxicity of internal cells, a culture. The principle of this study is that the yellow MTT reagent (3-(4,5-dimethylthiazolyl-2)-2,5-diphenyltetrazolium bromide) is reduced to a purple color by metabolically active cells. The final reagent results are then examined quantitatively using a spectrophotometer. The MTT assay test was performed on day 1, day 3, day 5 and day 7. Trypsinization was performed to remove the ASC layer on the culture flask. Each well in a 32-well microtiter tissue plate was filled with ASC suspension at a cell density of 8 × cells per well, the cell culture medium was then incubated for 24 h. Well, 4 repetitions were performed for each treatment group. After incubation for 1, 3, 5 and 7 days in a 5 % incubator at 37 °C. CO2At the end of the incubation, the medium in each well was discarded, washed with up to 100 μL of PBS and each well was given 100 μL of MTT, incubated at 37 °C for 4 h, then 200 μL of DMSO was added per well. Incubate again at 37 °C for 30 min. Living cells react with MTT (3-(4,5-dimethylthiazol-2yl)-2,5-diphenyltetrazolium bromide). The number of live cells turned blue with formazan, while the dead cells did not produce a blue color. The absorbance of formazan was measured spectrophotometrically using an ELISA reader with a wavelength of 570 nm. The darker the color, the higher the absorption value and the greater the number of living cells.

### 2.5 Alizarin red staining assay

In brief, the control and experimental groups were evaluated for calcium production on days 7, 14, and 21 of treatment using staining with alizarin red solution, a dye that binds calcium salts. Indeed, Alizarin red is an anthraquinone derivative used to identify osteocytes containing calcium in Adipose-Derived Stem Cell cultures. Cells were washed once with PBS and fixed in phosphate for 20 min and the supernatant was discarded. Cells were washed twice with PBS then fixed with neutral buffered formalin (10 %) for 30 min and washed with PBS. Alizarin Red Staining (40 mM in deionized water, pH 4.2) was added to the cells and incubated for 45 min in the dark. ASCs were washed four times and then PBS was added. For quantification, the stained cells were dissolved in 10 % acetic acid and the absorbance of the solution at a wavelength of 405 nm was calculated using a microplate reader.

### 2.6 Cell culture

Adipose Stem Cells (ASCs) were isolated from visceral fat tissue of young male rabbits. ASCs were washed using Phosphate Buffer Saline (PBS) and incubated with collagenase I 1.5 mg/mL, 30 mL (Invitrogen), 37 °C, for 30–45 min. The enzyme was inactivated by adding α-MEM containing Fetal Bovine Serum (FBS). Samples were centrifuged at 2000 rpm for 5 min and the cell pellet was resuspended in complete medium consisting of Dulbecco's Modified Eagle's Medium (DMEM; Gibco), 10 % Fetal Bovine Serum (FBS; Gibco), penicillin 100 units/mL, streptomycin 100 μg/mL, and Fungizone solution (Antibiotic-Antimycotic (100x) Gibco, USA) 0.25 μg/mL. The cells were planted into tissue culture polystyrene dishes (TCPS) measuring 25 cm^2^ and were incubated at 37 °C. After 72 h, cells were washed using PBS, α-MEM medium was added according to the capacity of the culture plate. The cells were kept in an incubator at 37 °C with a humidity of 5 % CO2. The medium was changed every two days until the cells reached 80–90 %. Characterization of ASCs was carried out using immunocytochemical examination, namely by staining anti-CD45, anti-CD73, anti-CD90, anti-CD105 (Sigma Aldrich®, USA).

### 2.7 Immunocytochemistry (ICC)

The monolayer cells were dissociated into single cells through trypsinization. Centrifugation was performed at 1600 rpm for 5 min. The cell pellet was resuspended in 1 ml of medium and seeded onto special glass slides at a volume of 20 μl. The glass slides were then placed in a box containing wet paper towels and incubated at 37 °C for 1 h. Fixation was carried out with 3 % formaldehyde for 15 min at room temperature. The slides were washed with PBS four times and allowed to dry. Blocking was performed with PBS containing 1 % serum for 15 min at room temperature. After washing with PBS four times, antibodies against Osterix, Osteopontin, RUNX2 and ALP were added and incubated at 37 °C for 45 min. The slides were washed with PBS four times, and excess water around the glass slides was dried with tissue paper. 50 % glycerin was dropped onto the glass slides, and the results were immediately observed under a fluorescence microscope at 40× magnification. Positive fluorescence results were observed, while negative results were not detected.

### 2.8 Statistical analysis

Data was analyzed using IBM SPSS. (Version 27.0, 2020. Armonk, NY: IBM Corp). Data normality was tested by Shapiro-Wilk test. Data was tabulated as mean ± standard deviation. One‐way ANOVA was utilized to determine the statistical significance; a P ≤ 0.05 was considered significant.

## 3 Results

The results of the toxicity test of the combination of nano suspension from purple leaf and hydroxyapatite showed an increase in cell proliferation at all concentrations. Descriptively, the results of the analysis show that the average proliferation test results with the highest percentage of live cells were 12.5 nm on day 1, 100 nm on day 3, and 200 nm on days 5 and 7.

ASCs characterizations were carried out using immunocytochemical examination. This test confirmed that the ASCs were mesenchymal derivatives. The results of immunocytochemical examination using an inverted and fluorescence microscope showed that the expression results of the CD 45 surface marker were negative, which was indicated by no green glow. The expression results of the surface markers CD90 and CD105 were positive as indicated by the green glow of ASCs.

### 3.1 Immunocytochemistry (ICC) result

**Fig. 1:**
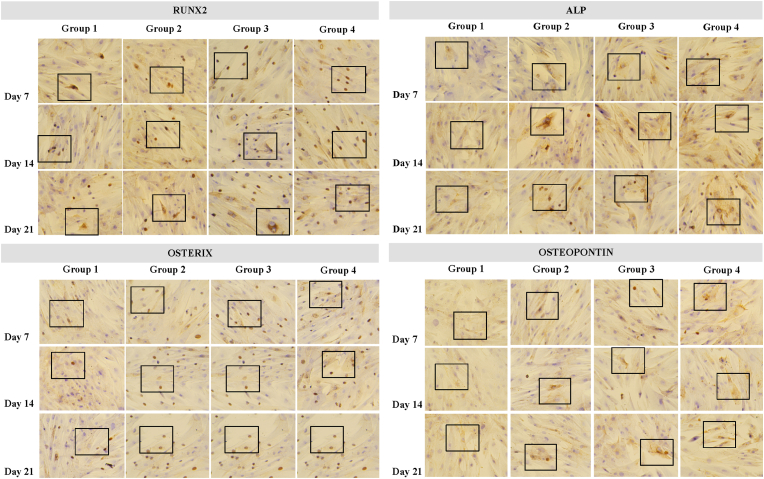
The ICC result of Runx2, ALP, Osterix and Osteopontin in 400x magnificient

**Fig. 2:**
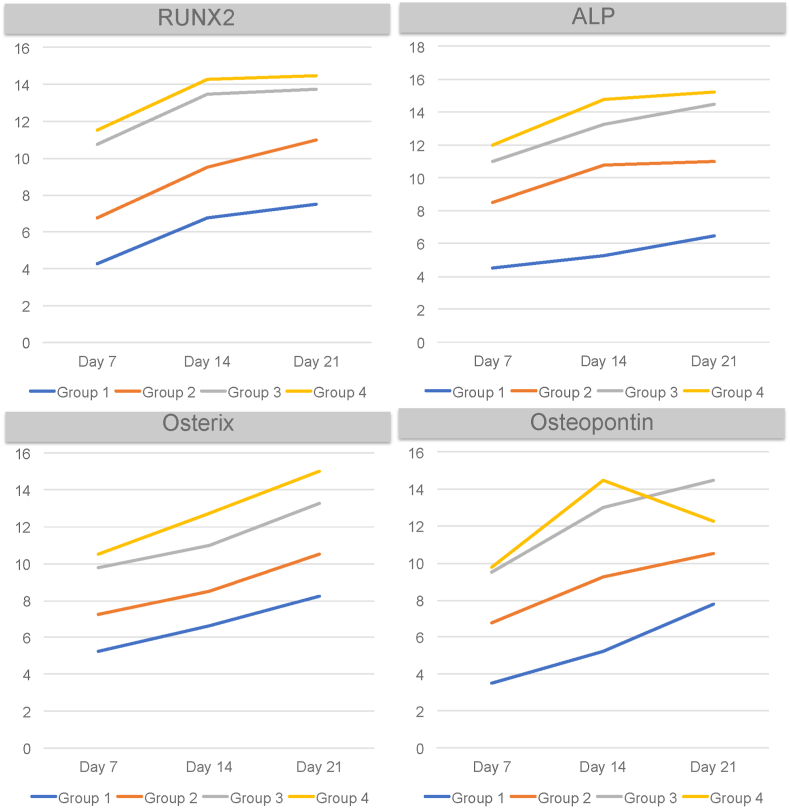
The number of ICC result in graphic.

The ICC result of Runx2, ALP, Osterix and Osteopontin in each group from day 7, day 14 and day 21 represented on Fig. 1, Fig. 2.

### 3.2 Alizarin red staining result

**Fig. 3:**
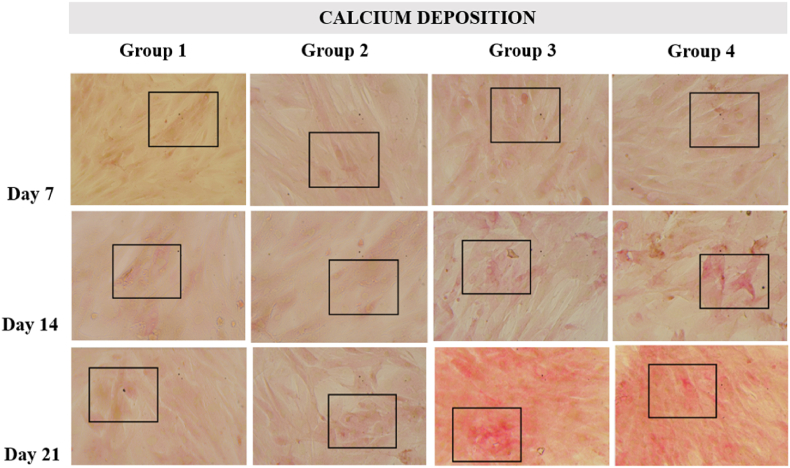
Alizarin Red S stained extracellular calcium deposition of the representative group. (For interpretation of the references to color in this figure legend, the reader is referred to the Web version of this article.)

**Fig. 4:**
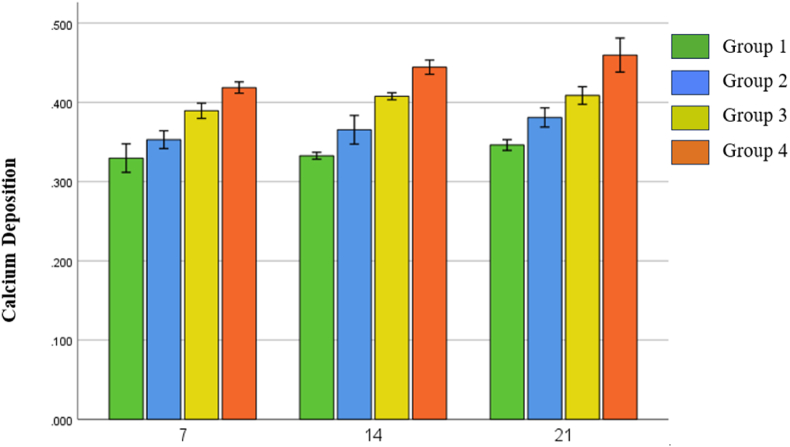
Average of calcium deposition on 7, 14, and 21 days.

Mineral depositions of ADSCs were detected by alizarin red staining following 7, 14, and 21 days of osteogenic induction with combination of nanosuspension (Fig. 3, Fig. 4).

## 4 Discussion

Graptophyllum pictum (purple leaves) contains non-toxic alkaloids, flavonoids, steroids, saponins, and tannins. Among these compounds, some can stimulate the expression of osteogenic transcription factors and markers through various signaling pathways, such as the Wnt and MAPK pathways, to promote osteoblast differentiation.^15^ Alkaloids and flavonoids in purple leaves possess anti-inflammatory and analgesic properties. Previous research indicates that purple leaves can stimulate ALP activity by 128 % in MC3T3-E osteoblast cells. Hydroxyapatite (HA) (Ca10(PO4)6(OH)2), an inorganic mineral constituent of human bone, can also be derived from animal bones. HA directly impacts bone defects by inducing a favorable immune response and promoting new blood vessel formation in damaged bone tissue.^16^ HA activates osteoblasts and osteoclasts for bone remodeling by accelerating the differentiation of these cells.^17^

In this study, a cell migration test was carried out on Adipose Stem Cells using the Scratch assay method. Cell migration and proliferation are important processes that trigger the synthesis of new extracellular matrix and contribute to wound healing. Recruitment of mesenchymal stem cells to the wound area is necessary to prepare for osteoblast differentiation at the ossification stage.^18^ Based on the research results, ASCs cultured with a combination of purple leaf extract nanosupension and hydroxyapatite in osteogenic medium showed cell migration ability as indicated by a significant increase in the percentage coverage of the scratch gap area from day to time. This may be caused by the presence of calcium ions in the nanosuspension which supports migration and proliferation. The EDX test shows a high number of Ca^2+^ ions in the combination of purple leaf extract nanosuspension and hydroxyapatite, where these ions can help increase the pH of the surrounding area and have a positive influence on the healing process.^19^

Based on the research results on days 7, 14, and 21, an increase in the mean expression of Runx2 was observed on day 21 in the group 4. This group had the highest hydroxyapatite (HA) content. HA is a biomaterial that can enhance osteoblast differentiation by increasing Runx2 expression through the activation of extracellular signal-regulated kinase (ERK), p38, Wnt, and bone morphogenetic protein 2 (BMP-2) pathways.^9^ Based on the post hoc analysis results for days 7, 14, and 21, significant differences were found between the group 1 and the treatment groups 3 and 4. The administration of groups 3 and 4 affected Runx2 expression on days 7, 14, and 21. In the treatment group 3 compared to the treatment group 4, post hoc analysis did not show significant differences. However, descriptive results indicated an increase in the mean expression of Runx2 in the latter treatment group.

The post hoc analysis results on days 7, 14, and 21 between the treatment group 3 and the group 4 showed no significant differences. This indicates that the potential of each concentration, whether group 3 or group 4, is almost the same. However, post hoc analysis results showed that on days 7 and 14, there was a significant difference between the group 3 and the group 4 on day 21. Therefore, it can be said that the concentration of the nanosuspension extract of purple leaves and HA bovine bone and the treatment day can influence the expression of the osterix marker. This is consistent with previous research, which found that the concentration of an extract could affect the number of osteoblast cells.^20^ The observation period from day 7, 14 to 21 in this study showed an increase in the average expression of osteopontin in the group 3 from day 7 to day 21. However, in the group 4, there was an increase in the average expression of osterix from day 7 to day 14, followed by a decrease on day 21. Nevertheless, post hoc analysis results on day 21 indicated no significant difference between the group 3 and the group 4. Thus, it can be said that although the average expression of osteopontin in the group 4 decreased on day 21, there was no significant difference compared to group 3.

The expression of the osteopontin marker between the group 1 and the group 3 showed significant differences on days 7, 14, and 21. Similarly, there were significant differences between the group 1 and the group 4 on days 7, 14, and 21. The combination of nanosuspension extract of purple leaves and HA bovine bone helps to enhance the differentiation process of osteoblasts, as HA bovine bone can stimulate the proliferation of osteoblasts by activating mesenchymal cells.^21^ Post hoc analysis results show that in the group 3, there was a significant difference between days 7 and 21. The post hoc analysis also revealed a significant difference between the group 3 on day 7 and the group 4 on days 14 and 21. In the group 4, there was also a significant difference between days 14 and 21.

In all treatment groups, the mean ALP expression increased on day 21. Based on the post hoc analysis, significant differences were found between the group 1 and the treatment groups (group 3 and 4), on days 7 and 14. These results indicate that although ALP is ideally expressed after day 14, the addition of group 3 and 4 can increase ALP expression on days 7 and 14.^16^ The post hoc analysis results support that the administration of group 3 and 4 can enhance ALP expression. Significant differences were observed when comparing the negative control group on days 7, 14, and 21 with the treatment groups on day 21. This is consistent with the theory that the flavonoid content in purple leaves exerts osteogenic effects through the ERK pathway dependent on estrogen (ER).^22^

Purple leaves have a stimulating effect on ALP (Alkaline Phosphatase) activity in osteoblast cells. The flavonoid content in purple leaves exerts osteogenic effects through the estrogen-dependent ERK pathway. Estrogen receptors (ER) bind flavonoids, which subsequently activate the ERK signaling pathway, leading to the upregulation of osteogenic genes and proteins essential for bone formation and mineralization.^22^ Previous research has shown a relationship between dose and biological response, where higher concentrations of active ingredients cause more pronounced biological effects.^23^

Overall, the trend in the amount of calcium deposition increased from days 7, 14 and 21 and the amount of calcium deposition in the administration of the nanosuspension (Group 1 and 2) was higher than that of the untreated (Group 1 and 2), with the highest levels in the group 4. This is likely influenced by the presence of active compounds in purple leaf extract such as polyphenols and alkaloids which play a role in helping the osteogenic differentiation process of ASCs.^24^ One of the phenolic contents that was successfully identified in the combination of purple leaf extract nanosuspension and hydroxyapatite is salicylic acid. Salicylic acid is a simple phenolic compound that is naturally found in plants. This compound is a precursor of Acetylsalicylic Acid or better known as aspirin. Acetylsalicylic Acid itself has been shown to increase osteoblast differentiation in human Dental Pulp Mesenchymal Stem Cells (hDPMSC) by activating the MAPK signaling pathway.^25^

The results obtained from this study confirm that the combination of purple leaf extract nanosuspension and hydroxyapatite as a biomaterial for bone tissue engineering can be a scaffold for bone tissue regeneration. The combination of purple leaf extract nanosuspension and hydroxyapatite has characteristics that can increase the osteogenic differentiation of ASCs in vitro. However, a more detailed mechanism of the effect of ASCs regulated by the combination of purple leaf extract nanosuspension and hydroxyapatite is still unknown, so further research is needed.

This principle applies to the use of purple leaves extract in osteogenic differentiation, where a 2 % concentration produces a more significant response compared to a 1 % concentration. These findings are consistent with the theory from previous studies, which demonstrated that increasing the concentration of bioactive compounds generally enhances biological activity due to stronger activation of cellular pathways and receptor interactions.

## Patient consent

This research not use patients.

## Source funding

This research is funded by Lembaga Penelitian dan Pengabdian Masyarakat, Universitas Airlangga under the scheme of Airlangga Research Fund Batch 2 (Ref. No: 1532/UN3.LPPM/PT.1.02/2023).

## Declaration of competing interest

The authors declare that they have no known competing financial interests or personal relationships that could have appeared to influence the work reported in this paper.
